# The Diet of Preschool Children in the Mediterranean Countries of the European Union: A Systematic Review

**DOI:** 10.3390/ijerph13060572

**Published:** 2016-06-08

**Authors:** Luís Pereira-da-Silva, Carla Rêgo, Angelo Pietrobelli

**Affiliations:** 1 NOVA Medical School, Universidade Nova de Lisboa, Lisbon 1169-056, Portugal; 2 Pediatric Department, Hospital Dona Estefânia, Centro Hospitalar de Lisboa Central, Lisbon 1169-045, Portugal; 3 Faculty of Medicine, Porto University, Porto 4200-319, Portugal; carlambssrego@gmail.com; 4 Center for Health Technology and Services Research (CINTESIS), Porto 4200-3129, Portugal; 5 Child and Adolescent Service, Hospital CUF Porto, Porto 4200-180, Portugal; 6 Pediatric Unit, Verona University Medical School, Verona 37134, Italy; angelo.pietrobelli@univr.it; 7 Pennington Biomedical Research Center, Baton Rouge, LA 70808, USA

**Keywords:** dietary intake, European Union, Mediterranean countries, Mediterranean dietary pattern, overweight and obesity, preschool children

## Abstract

This systematic review discusses data on the dietary intake of preschool children living in the Mediterranean countries of the European Union, including the comparison with a Mediterranean-like diet and the association with nutritional status. Specifically, data from the multinational European Identification and Prevention on Dietary and life style induced health effects in children and infants (IDEFICS) study and national studies, such as the Estudo do Padrão Alimentar e de Crescimento Infantil (EPACI) study and Geração XXI cohort in Portugal, ALimentando la SAlud del MAñana (ALSALMA) study in Spain, Étude des Déterminants pré-et postnatals précoces du développement et de la santé de l’ENfant (EDEN) cohort in France, Nutrintake 636 study in Italy, and Growth, Exercise and Nutrition Epidemiological Study in preSchoolers (GENESIS) cohort in Greece, were analyzed. In the majority of countries, young children consumed fruit and vegetables quite frequently, but also consumed sugared beverages and snacks. High energy and high protein intakes mainly from dairy products were found in the majority of countries. The majority of children also consumed excessive sodium intake. Early high prevalence of overweight and obesity was found, and both early consumption of energy-dense foods and overweight seemed to track across toddler and preschool ages. Most children living in the analyzed countries showed low adherence to a Mediterranean-like diet, which in turn was associated with being overweight/obese. Unhealthier diets were associated with lower maternal educational level and parental unemployment. Programs promoting adherence of young children to the traditional Mediterranean diet should be part of a multi-intervention strategy for the prevention and treatment of pediatric overweight and obesity.

## 1. Introduction

The “Mediterranean diet” is not a homogenous and exclusive diet model in the 16 countries situated around the Mediterranean Sea [1]. Nevertheless, a Mediterranean diet-like pattern is common in Mediterranean countries, and is based on a set of healthy dietary habits that include relatively high consumption of unprocessed foods and plant foods, such as vegetables, legumes, grains, nuts, fresh and seasonal fruit, breads, and unrefined cereals. The pattern is also associated with moderate to high intake of fish, moderate intake of dairy products (mostly cheese and yogurt), low intake of saturated fatty acids, and higher intake of unsaturated fatty acids, with olive oil as the main source of fat [1]. Despite this common dietary pattern, differences in culture, ethnic background, religion, economy, and agricultural production have resulted in different types of diets across the Mediterranean region [2]. Food consumption is affected by different factors, including food availability, accessibility, and choices, which in turn may be influenced by geography, demography, socioeconomic status, urbanization, globalization, marketing, and consumers [2].

Greater adherence to a Mediterranean-like dietary pattern is associated with a significant improvement in health status, especially reductions in the risk of developing metabolic syndrome and major chronic morbidities [3]. In the pediatric population, the Mediterranean Diet Quality Index (KIDMED), in association with obesity indicators, has been used to assess adherence to a Mediterranean-like dietary pattern; an inverse association between this pattern and childhood overweight with short- and long-term health implications has been described [4,5,6].

The rising availability of energy-dense foods (EDF) is likely to be one of the major drivers of the obesity epidemic in European countries [7]. The real price of EDF has reached its lowest in many European countries, although increasing availability has not always been accompanied by an improvement in quality [7]. In particular, agricultural policies in the European Union have encouraged the production of sugar, oils, and meat at low cost through subsidies and other measures, compared with the limited market supply of fruit and vegetables [7]. Recent data may be particularly useful in planning strategies and policies to correct currently inadequate eating practices and unbalanced diets. For instance, the energy intake of young children in Greece was reported to have increased in just a decade [8,9].

More investigations have been dedicated to studying the diet of children less than 12 months of age compared to that for the diet of older children. After 1 year of age, growth assessment and nutritional counseling by doctors and other health professionals become less frequent and the diet of the children is guided more by family, caregivers, and kindergarten [10] than healthcare professionals. In this context, the dietary practices and nutritional status of children deserve particular attention.

This systematic review is centered on recently published data on the dietary habits including the comparison with the Mediterranean-like diet, and weight status of children aged 1–5 years living in the Mediterranean countries of the European Union. We hypothesize that the reported changes in the dietary habits of the general Mediterranean population during the last decades, which have drifted away from the traditional Mediterranean diet-like pattern, have also occurred in toddlers and preschool children. We assume that the aforementioned agricultural policies in the European Union have an important influence on the dietary habits and food choices in the Mediterranean countries of the European Union.

## 2. Methods

### 2.1. Literature Search

Published multi-national and national cohort studies, surveys, and cross-sectional studies that investigated the dietary intake, including the comparison with a Mediterranean-like diet, and the related overweight and obesity of children in European Mediterranean countries, were systematically searched. A further refinement aimed to select studies published in the last decade (from January 2006 to January 2016) including children aged 1–5 years living in the Mediterranean countries of the European Union (from west to east): Portugal, Spain, France, Italy, Malta, Greece, and Cyprus. While Portugal is not geographically in the Mediterranean basin, it was included in the review because of its proximity to the Mediterranean region and the Mediterranean diet is a recognized cultural heritage of the Portuguese population [11]. The search was based on the electronic databases: Medical Literature Analysis and Retrieval System Online (MEDLINE) (source: PubMed; www.pubmed.com), Excerpta Medica Database (EMBASE) (www.embase.com), Web of Science (isiwebofknowledge.com), The Cochrane Library (source: The Cochrane Central Register of Controlled Trials; www.thecochranelibrary.com/), clinicaltrials.org, and Google Scholar (scholar.google.com). Relevant keywords relating to the Mediterranean diet in combination with MeSH terms and text words (“Mediterranean diet” or “diet” or “dietary pattern” or “dietary habits” or “Mediterranean countries” or “European Union” or “adherence” or “score”) were used in combination with words relating to weight status (“overweight” or “obesity”) and age group (“toddlers” or “preschool children” or “children”). The search strategy had no language restrictions. References from the extracted articles and reviews were also consulted to complete the data collection.

### 2.2. Quality Assessment

Study quality was assessed according to the following criteria: (1) number of study participants; (2) instruments used to assess the dietary intake, and/or dietary quality; (3) instruments used to assess the adherence to a Mediterranean-like diet; (4) criteria used to assess overweight and obesity; (5) duration of follow-up in prospective studies; and (6) adjustment for potential confounders.

### 2.3. Ethical Statement

The study was conducted in accordance with the Declaration of Helsinki, and the protocol was approved by the Ethics Committee of the Centro Hospitalar de Lisboa Central, Lisbon, Portugal (Project ID 340).

## 3. Results

Two multi-national European studies that included the Mediterranean countries of European Union, investigating dietary habits in comparison to a Mediterranean diet-like pattern, and weight status of preschool children, were identified: the Identification and Prevention on Dietary and Lifestyle-Induced Health Effects in Children and Infants (IDEFICS) study, a population-based survey [12], and a study collecting data from four representative European birth cohorts [13] (Table 1). In this regard, five studies nested within the IDEFICS study were analyzed separately [14,15,16,17,18] (Table 1).

National studies investigating the dietary habits and nutritional status of preschool children living in the Mediterranean countries of the European Union were identified as follows: in Portugal, the Estudo do Padrão Alimentar e de Crescimento Infantil (EPACI) a representative cross-sectional study [19,20] and five prospective or cross-sectional studies nested within the Geração XXI birth cohort [21,22,23,24,25]; in Spain, the ALimentando la SAlud del MAñana (ALSALMA) cross-sectional study [26]; in France, one national cross-sectional survey [27] and one study nested within the Étude des Déterminants pré- et postnatals précoces du développement et de la santé de l’Enfant (EDEN) mother-child cohort [28]; in Italy, one single-center birth cohort [29] and the cross-sectional Nutrintake 636 study [30]; in Malta, one cross-sectional study [31]; and in Greece, three cross-sectional studies nested within the Growth, Exercise and Nutrition Epidemiological Study in preSchoolers (GENESIS) cohort [9,32,33] (Table 2).

### 3.1. Dietary Intake in Comparison with the Mediterranean-Like Diet

For the IDEFICS study, 16,228 European children aged 2–9 years in 2007–2008 were recruited [12] to assess and compare dietary habits prevalent in Mediterranean Europe (Italy, Spain, and Cyprus), North Europe (Estonia and Sweden), and Central Europe (Belgium, Germany, and Hungary). Although this study was not designed to provide a representative sample for each country, it contains some unique national data for the participants. In addition, it is one of the largest European children’s cohort established to date, comprising standardized measurements from eight centers across different European countries with a 2-years follow up for prospective analyses [17]. The associations between validated proxy-reported energy intake, daily food intake, EDF and body mass index (BMI) z-scores were investigated in a subsample of 8551 pre-school children from the IDEFICS study [14]. Dietary intake during the previous 24 h was assessed using the “Self-Administered Children and Infant Nutrition Assessment” and country-specific food composition tables were used to match simple foods or pan-European homogeneous multi-ingredient food items. It was observed that proxy-reporters are subject to misreporting, in particular for children with higher BMI levels, and that energy intake is a more important predictor of unhealthy weight development in children than the daily food intake [14] (Table 1). Another study from the IDEFICS [15] used 24-h dietary recalls *plus* school meal assessments to describe energy density of dietary intake. Pre-school Mediterranean children consumed mean food densities of 1.81 Kcal/g from exclusively solid foods and of 1.27 Kcal/g from solid foods *plus* energy-containing beverages; the daily intake from beverages was 284 Kcal [15] (Table 1).

The adherence to a Mediterranean-like dietary pattern was examined in a subsample of 7940 children from the IDEFICS study using the available 24h dietary recalls [16]. Responses to a baseline parental questionnaire on diet together with anthropometric measures were collected. The validated self-administered Children’s Eating Habits Questionnaire assessed frequency of consumption of 43 foods and other diet-related habits [34,35]. Since the questionnaire did not include quantitative intakes, adherence to a Mediterranean-like diet was assessed by a food frequency-based Mediterranean Diet Score (fMDS). This score was calculated based on the age- and sex-specific population median intakes of six food groups (vegetables and legumes, fruit and nuts, cereal grains and potatoes, meat products, and dairy products) and the ratio of unsaturated to saturated fats; fish and seafood was consumed by 10% of the population. The demographic and socioeconomic characteristics (parental education and income) of children showing high (>3) *vs.* low (≤3) fMDS levels were examined. In Mediterranean countries, the percentages of a high adherence to a Mediterranean-like diet in preschool (<6 years) children were 55.0% and 55.9% for girls and boys, respectively, in Italy (the highest rates among the participant countries), 37.1% and 32.3% in Spain, and 29.5% and 33.9% in Cyprus (the lowest rates among the participating countries) [16] (Table 1). In the whole sample (preschool and school children), higher adherence to a Mediterranean-like dietary pattern was not associated with living in a Mediterranean region [16].

Another analysis from the IDEFICS study assessed the prevalence of overweight and obesity in 7940 European children aged 2–10 years [18]. The examination program included standard anthropometric measures such as weight, height, skinfolds, waist circumference, and bioelectrical impedance measured by trained professionals using accurate and precise portable instruments. According to the recently updated International Obesity Task Force (IOTF) 2012 cut-offs [36], a higher prevalence of overweight and obesity was found in Mediterranean countries (25.0% and 17.4% in Italy, 15.5% and 7.2% in Cyprus, and 14.3% and 10.0% in Spain, respectively) than in other European countries. Overall, population groups with low income and/or lower education levels showed the highest prevalence of obesity [18].

Using the fMDS, the association between a Mediterranean-like diet and obesity was assessed in a subsample of 9114 children from the IDEFICS study using the available anthropometric measurements [17]. The IOTF 2000 cut-offs [37] were used to define overweight and obesity. In the pooled sample (preschool and school children), low fMDS levels were associated with overweight including obesity (OR = 0.85, 95% CI: 0.77–0.94) and percentage of fat mass (β = −0.22, 95% CI: −0.43–−0.01) independent of age, sex, socioeconomic status, and physical activity. High fMDS levels at baseline protected against increases in BMI (OR = 0.87; 95% CI: 0.78–0.98), waist circumference (OR = 0.87; 95% CI: 0.77–0.98), and waist-to-height ratio (OR = 0.88; 95% CI: 0.78–0.99) with a similar trend for percentage of fat mass (*p* = 0.06). Parental education and high parental income were determinants of high adherence, as indicated by high fMDS levels (OR = 1.21; 95% CI: 1.11–1.32; OR = 1.07; 95% CI: 0.98–1.17, respectively) [17] (Table 1). Some limitations were acknowledged by the authors. A single recall may not necessarily reflect habitual intakes and misclassifications may have occurred especially in case of non-daily consumed food groups like fish. Low income groups were not equally distributed across all centers, and consequently this could have limited the possibility to adjust for confounding socioeconomic status. The questionnaire used was limited to 43 items, thus leading to a risk in underestimating intakes. Finally, the fact that the study was not designed to assess portion sizes might have led to further over- or underestimation [16,17].

The influence of early feeding practices on fruit and vegetable intake was assessed in preschool children in four representative European birth cohorts [13]: 7269 children in the British Avon Longitudinal Study of Parents and Children (ALSPAC) study, 1302 in the French EDEN study, 556 in the Portuguese Generation XXI Birth Cohort, and 800 in the Greek EuroPrevall study. Fruit and vegetable intake was assessed in each cohort using a food-frequency questionnaire (FFQ), and associations between early feeding practices, such as breastfeeding and timing of complementary feeding, and fruit and/or vegetable intake in 2–4 years old children were analyzed by using logistic regressions, separately for each cohort. It was found that breastfeeding a child for a longer period after birth was consistently related to higher fruit and vegetable intake. In the Mediterranean cohorts, mean fruit and vegetable intakes (servings/day) were 1.3 and 1.1 in the French EDEN and 1.1 and 0.7 in Greek-EuroPrevall studies, respectively, at 2-years of age; and 1.7 and 3.3 in the Generation XXI Cohort at 4-years of age [13] (Table 1). Sufficient intake of fruit and vegetables is particularly relevant, since it has been reported to be associated with a reduced risk of chronic diseases and body weight management, although the exact mechanism is unknown [38,39]. Because fruit and vegetables are high in water and fiber, incorporating them in the diet can reduce energy density, promote satiety, and decrease energy intake. A particularly effective strategy for weight management may be coupling advice to increase intake of whole fresh fruit and vegetables with advice to decrease energy intake [39].

### 3.2. Data from Specific Countries

#### 3.2.1. Portugal

Portugal is the westernmost country in South Europe and is geographically not in the Mediterranean area. Instead, Portugal is bordered to the west and south by the Atlantic Ocean and to the east and north by Spain. However, a Mediterranean diet is a recognized cultural heritage of the Portuguese population [11], with ancestral influence from Mediterranean neighbors and migrants.

In 2012, the EPACI cross-sectional study evaluated the feeding pattern and growth of 2232 Portuguese children aged 12–36 months [19]. This national representative study was carried out in 128 primary health care centers by trained professionals. Food pattern was assessed using a food-frequency questionnaire (FFQ) and dietary intake was assessed using a 3-day food-diary (two week-days and one weekend day; Food Processor SQL). Anthropometry was performed using accurate and precise portable instruments and nutritional status was characterized according to WHO criteria [19]. It was found that 69% of daily meals were eaten at home, 12% at kindergarten, and 10% at grandparent’s house. Most children frequently ate fresh fruits (93%) and vegetables (99%) daily; most vegetables were consumed in the form of pureed soup (95%). The consumption of fruit and vegetables was higher in kindergarten than at home. A high prevalence of daily consumption of sugared beverages, such as commercialized juices, ice tea, and cola (17%), and of sugared desserts (10%), with high-energy density and low nutritional value, was found. Nearly half of all children (33.8% and 70.5% at 12–24 and 24–36 months of age, respectively) consumed higher than recommended energy intake [40]; and the mean daily protein intake was 4.5 g/Kg, four times more than the recommended dietary allowances (RDA) [40]. The mean (SD) percentage of total energy value (%TEV) was 49.9% (3.6) from carbohydrates, 28.5% (2.7) from fat, and 18.2% (1.7) from protein. The food groups contributing most to the total energy intake were dairy products (28%), cereals (excluding rice and pasta) (15%), and meat (13%). The food groups contributing most to total protein intake were dairy products (36%), meat (28%), and fish (13%). Of note, 87.3% of children exceeded the upper recommended limit (1500 mg) for daily sodium intake, the mean being 1843 mg. The prevalence of inadequacy was near zero for the majority of micronutrients, except for vitamin E in all sample (89%; 95% CI: 0.86–0.92), and folate (20.5%; 95% CI: 0.17–0.24) and vitamin B1 (3.0%; 95% CI: 0.02–0.05) during the second year of life [18,19]. In this study, high BMI was found at early ages, with overweight in 24.9%, and obesity in 6.5% [19,20].

Several important longitudinal studies were derived from Geração XXI, the largest Portuguese birth cohort that enrolled 8647 infants born in 2005–2006 in North Portugal [23]. Dietary intake was assessed using an FFQ *plus* food diaries. In the Geração XXI studies, a common anthropometric method was used by trained professionals. Either recumbent length was measured to the nearest centimeter with a length measuring board or height was measured to nearest centimeter using a wall stadiometer when children were able to stand alone. Body weight was measured in light clothing to the nearest 0.1 kg using a digital scale [23]. In a Geração XXI study it was found that at 4-years of age the children of the Geração XXI cohort frequently had daily consumption of fruit (86%) and vegetables (92%), typically in the form of pureed vegetables [21]. However, an important proportion of these children ate cakes and candies at least once a day (65%), and drank sugared beverages at least once a day (52%); 73% consumed salty snacks (pizza, hamburger, French fries and packed snacks) 1 to 4 times/week [21]. The main sources of saturated fatty acids were dairy products (33.4%), meat (20.7%), savory pastries, and sugared desserts (14.7%). According to dietary reference intakes (DRI) and estimated average requirement (EAR) cut-offs, the prevalence of insufficiencies for the majority of micronutrients was low, except for insufficient intake of vitamin D (100%), vitamin E (98.6%), folate (21.5%), and calcium (7.0%) [21]. They also found excessive sodium intake (99%), with salt added to soups responsible for 1/3 of the sodium intake. The 4-year-old children had high BMI; 13.8% were overweight and 6.2% were obese. Most data found in these children are consistent with the findings in the 1–3 year-old children of the EPACI study, particularly the frequent consumption of fruit and vegetables with excessive energy and sodium intake and a high prevalence of overweight and obesity [19,20]. Excessive sodium intake from a young age is a matter of concern. The development of taste preferences occurs during childhood and salt intake is often associated with the consumption of sugar-sweetened beverages, predisposing children to weight gain [41]. A blood pressure tracking phenomenon has also been recognized [41].

The association of socioeconomic status and family structure with consumption of EDF at 2-years of age was assessed in a sample of 808 children from the Geração XXI cohort using information on food consumption [22]. A FFQ was administered to parents and four groups of EDF were defined: soft drinks (sweetened drinks), sweets (chocolate and candies), cakes (creamy and non-creamy cakes and sweet pastries), and salty snacks (crisps, pizzas, and burgers). High socioeconomic status was associated with lower EDF consumption, mainly soft drinks and sweets. This influence was not only from the parental background but also from the preceding generations [22]. Children with older siblings were more likely to have daily consumption of any energy-dense food [22]. Nevertheless, EDF intake was much lower than found in similarly aged children in other westernized countries [42,43].

The association between EDF consumption at 2 years of age and the consumption of foods and diet quality at 4-years of age was evaluated in a sample of 705 children from the Generation XXI cohort [23]. The aforementioned four EDF groups [22] were defined and a healthy eating index was developed using the WHO dietary recommendations for 4-year-old children [44]. It was found that EDF consumption at a young age was negatively associated with the diet quality at a later age. Higher intakes (≥median) of soft drinks (incidence rate ratio (IRR) = 0.74, 95% CI: 0.58–0.95), salty snacks (IRR = 0.80, 95% CI: 0.65–1.00), and sweets (IRR = 0.73, 95% CI: 0.58–0.91) in 2 year old children were associated with lower consumption of fruit and vegetables in 4 year old children (≥5 times/day) [23].

The association between EDF consumption at 2 years of age and BMI at 4 years of age was determined in a sample of 589 children from the Generation XXI cohort, using a cross-lagged panel design [24]. Although the hypothesized association was not found, both the consumption of EDF (β = 0.522; 95% CI: 0.432–0.612) and BMI z-scores (β = 0.747; 95% CI: 0.688–0.806) tracked over time [24].

Maternal perceived responsibility and child-feeding practices were assessed in 4122 4-year-old children from the Generation XXI cohort [25] using a combined version of the Child Feeding Questionnaire [45] and the scales of overt and covert control [46] self-administered to mothers, with a version validated and adapted to Portuguese [47]. It was found that children whose mothers had higher levels of covert control, monitoring, and restriction were less likely to consume fruits and vegetables below recommendations and EDF above tolerable limits [25]. In addition, higher pressure to eat was associated with a higher possibility of children consuming dairy above recommendations [25]. While this result is comparable with another European study [48], others have shown restriction to be associated with subsequent higher consumption of palatable snacks in the absence of hunger [49]. This may be explained by a higher preference for the restricted food and higher responsiveness to external cues such as the presence of freely available palatable foods [49]. There is the possibility that some level of parental control, probably a moderate level, may be beneficial [50].

#### 3.2.2. Spain

The ALSALMA cross-sectional study analyzed the nutritional patterns of 1701 children under 3 years of age and compared the results with current recommendations [26]. The parents completed a dietary diary of food intake for their children for 4 non-consecutive days. The mean energy intake was 123%–124% higher than the DRI for 13–36 month old children. The mean percentage of protein intake increased with age, being 370% and 441% higher than adequate intake for 13–24 and 25–36 months, respectively [26]. These results are analogous to those reported in other European countries in similar age groups [51]. Using multiple linear regression analysis, it was found that higher percentages in daily intake of proteins and carbohydrates and lower percentage of total lipids were significantly related to a greater BMI, regardless of energy intake [26]. The proportions of children with deficient micronutrient intakes, that is, below the EAR [52,53], were 81.7% and 92.1% for vitamin D; 39.3% and 53.4% for vitamin E; 12.5% and 14.8% for folic acid; 10.1% and 5.5% for calcium; and 27.1% and 31% for iodine, for 13–24 and 25–36 months of age, respectively [26].

An analysis nested within the IDEFICS study described the energy density of the dietary intake of 8551 children using 24-h dietary recalls [15]. Despite being the lowest values among the participating countries, the subsample of 289 children aged 2–5 years in Spain consumed daily mean energy densities of 1.67 Kcal/g exclusively from solid foods and of 1.16 Kcal/g from solid foods *plus* energy-containing beverages, in which the mean energy intake from beverages contributed with 307 Kcal per day [15].

A cross-sectional survey evaluated in Spain the relevance of the main nutritional problems perceived by 155 pediatricians in children under 3 years of age [10]. Parents were considered to have the main influence regarding their children’s nutritional health; however, their concerns with this issue significantly reduced as the children grew older. In addition, the proportion of children who received nutritional counseling reduced from 88% at 0–6 months to 61% at 24–36 months [10].

#### 3.2.3. France

A national cross-sectional survey conducted in France in 2005 assessed the energy and nutrient intake and adequacy of diet in 706 children aged 1 to 3 years [27]. The weight of food records individually noted for 3 consecutive days were converted into energy intake and intake of 24 nutrients, according to food composition databases for 1260 standard foods and all formulae and specific baby foods manufactured and marketed in France in 2005. Mean daily energy intake was slightly higher than the EAR up to 7 months of age, but it became lower the EAR after 1 year of age. Globally, it was found that diet was adequate for a large proportion of toddlers and satisfied most of their nutritional requirements. Mean daily intake of energy, protein, fat, and carbohydrate were considered adequate, whereas sodium, calcium, magnesium, phosphorus, and B group vitamins were above the DRI. Mean sodium intake in particular was above the adequate intake for all age subgroups. Some children over 1 year of age had inadequate intakes of α-linolenic acid, vitamin E, vitamin C, iron, and zinc [27].

The first study in France assessing dietary patterns across toddlers and preschoolers was based on the EDEN mother-child cohort [28]. To estimate tracking between dietary patterns, a cross-section of 989 children from this cohort were recruited to collect dietary intake using an FFQ at 2, 3, and 5-years of age. Three dietary patterns in toddlers aged 2 years were identified as follows: “processed and fast foods”, positively correlating with intake of French fries, processed meat, carbonated soft drinks, chocolate, chips, cookies, pizza, fruit juice, meat, dairy desserts, and ice cream (in descending order); “guidelines”, characterized mainly by high frequency consumption of cooked vegetables, rice, fresh fruit, raw vegetables, low-fat fish, potatoes, ham, stewed fruit, and meat; and “baby foods”, positively correlating with baby foods, breakfast cereals, and stewed fruit [28]. Overall, moderate correlation coefficients were found between similar patterns assessed across the three ages, reaching correlation coefficients of 0.40 and 0.53 for “processed and fast foods” and “guidelines” dietary patterns, respectively [28]. The “processed and fast foods” pattern at 2, 3, and 5-years was inversely associated with maternal education and age, and positively associated with the presence of older siblings. The “guidelines” pattern at 2, 3, and 5 years was predicted by maternal education [28]. These findings suggest the emergence of dietary profiles, socially differentiated, early in life, a moderate tracking of dietary patterns from 2 to 5 years of age, and a significant influence of maternal education on the child’s diet [28]. Given the evidence that taste and food preferences are built upon repeated exposures to specific foods [54], these data give ground to encourage the promotion of healthy feeding practices and healthy dietary trajectories from early infancy.

#### 3.2.4. Italy

The dietary energy and macronutrient intakes were longitudinally analyzed up to 10 years of age in a birth cohort of 61 healthy Italian children [29]. In 1994, subjects were recruited randomly during their last trimester at a maternity unit in Milan, and the generalizability of this single-center study based on a small sample size can be questioned. Regarding the results for children at preschool age, at 1 year of age the mean (SD) total daily energy intake was 94 (27) Kcal/Kg and TEVs were 48.3% (6.8) from carbohydrate, 33.6% (5.1) from lipids, and 20.4% (3.5) from protein; at 5 years of age, the total daily energy intake was 119 (29) Kcal/Kg and TEVs were 57% (5) from carbohydrate, 32% (4) from lipids, and 15% (2) from protein. In a longitudinal analysis, boys had higher energy intake (*p* < 0.0001) and glycemic load (*p* < 0.0001) [29]. Compared with Italian recommended dietary allowances (RDA), protein intake was higher at all analyzed ages and energy intake was higher at 5 years of age [29].

In an analysis nested within the IDEFICS study, Italy participated with the greatest subsample (20.1%); 776 children aged 2–5 years in Italy consumed a daily mean energy density of 2.24 Kcal/g exclusively from solid foods and 1.61 Kcal/g from solid foods *plus* energy-containing beverages, providing the highest values among the participating countries [15]. The mean energy intake from beverages contributed 208 Kcal per day [15].

In 2012–2013, the NUTRINTAKE cross-sectional study compared the dietary intake of 6–36 month-old children living in north and south Italy [30]. Three-hundred and ninety children were recruited, 189 living in Milan (north) and 201 in Catania (south). Dietary intake was assessed using a 7 day weighed-food record. In the pooled sample, normal anthropometry and energy intake were found. However, high intake of proteins, simple carbohydrates, saturated fats, and sodium, and low intake of iron and fiber were found, compared with Italian reference values. Anthropometry, energy, and macronutrient intakes were similar in Milan and Catania, but iron intake was 27% lower and fiber intake 16% higher in Milan than in Catania [30].

#### 3.2.5. Malta

No recent national representative data are available on food consumption patterns in young children in Malta. The associations between parenteral and childhood obesity and between maternal awareness of public health promotion on healthy eating and parenteral and childhood obesity were evaluated in 200 groups of 3-year-old children and their parents, randomly selected from the Maltese public registry list [31]. The study was carried out at one public health care center using face-to-face interviews. Parents were shown leaflets issued by the Department of Health Promotion on “Positive Health Behaviour” and “Healthy Eating Habits” and asked whether they were aware of the leaflets. Health promotion awareness was assessed by rating the level at which mothers knew, read, remembered, and practiced health promotion messages regarding nutrition. In this study, weight-for-height percentiles were used to define overweight (between 75th and 97th percentiles) and obesity (>97th percentiles). In univariate analysis, a statistically significant association was found between childhood obesity and obesity in either of the parents (r = 0.2 both for mothers and fathers, *p* < 0.001). Full maternal awareness of healthy eating promotion appeared to have a protective effect against the development of childhood obesity/overweight (OR = 0.38, 95% CI: 0.20–0.70) [31].

#### 3.2.6. Greece

Several studies on the dietary intake of toddlers and preschoolers in Greece were nested within the GENESIS cohort [55]. In 2003–2004, a cross-sectional study randomly selected a representative sample of 2374 children aged 1–5 years from five Greek counties, from the GENESIS cohort [9]. Dietary intake was assessed for 2 consecutive weekdays weighing and recording all foods consumed and for one weekend day by an interview with the parent/guardian. The nutrient adequacy of the diets was assessed in these children, according to the method recommended by the Institute of Medicine [56]. In relation to energy intake, children’s requirements were expressed in estimated energy requirements [52]; for nutrients with an EAR, the proportion of children with usual intakes less than that value was estimated [56,57]; for nutrients with established tolerable upper intake levels, the proportion of children with usual intake from food exceeding those levels was calculated; and the proportion of children with usual intakes outside the acceptable macronutrient distribution ranges (AMDR) for fat, protein, and carbohydrate intakes as a percentage of energy intake was examined [52]. A common method for anthropometry was used in all GENESIS studies; the instruments used were highly accurate and precise, yet sufficiently portable to be carried to nursery schools, where the measurements took place; measurements were taken and recorded by two well-trained team members. The anthropometric measurements included body weight, recumbent length/standing height (children older than 2 years of age), head, waist, hip and right arm circumferences, and skinfolds (triceps, biceps, subscapular, and suprailiac). Weight-for-length cut-offs were used to classify children up to 24 months old as “at risk of being overweight” (≥85th and <95th percentile) and “overweight” (>95th percentile), whereas in children older than 24 months the same classifications were based on similar BMI cut-offs [9]. The prevalence of “at risk of being overweight” and “overweight” in the pooled sample was 16.7% and 16.4%, respectively, and the mean usual energy intake was 117% of the mean estimated energy requirement. The “at risk of being overweight” and “overweight” children had significantly higher mean daily energy intakes (1434 and 1445 Kcal) than their normal-weight counterparts (1386 Kcal). In addition, “overweight” children consumed more energy from total fat and saturated fat and less from carbohydrate [9]. The usual protein intake was within the AMDR, but 21.0% of children had intakes lower than the AMDR for carbohydrate and 59.5% higher than the AMDR for fat [9]. The estimated prevalence of inadequacy was found to be between 10% and 25% for niacin, vitamin E, and folate, and usual intakes exceeding the tolerable upper intake levels were recorded for zinc and copper [9].

The diet quality of preschoolers in Greece and related sociodemographic were assessed in a representative sample of 2287 children aged 2–5 years from the GENESIS cohort [32]. Dietary intake data was obtained using a combination of techniques comprising weighed-food records, 24-h recalls, and food diaries. The “Healthy Eating Index” (HEI) score was calculated summing the individual scores (0 to 10) assigned to each one of 10 index components. About 80% of children had a poor diet (low HEI score) associated with low fruit, vegetable, and grain intake and high saturated fat intake. In multiple linear regression, poor diet was associated with lower levels of physical activity, lower maternal educational level, and unemployment status [32]. Similar to the Revised Children’s Diet Quality Index [58], the HEI seems to be a good index to assess diet quality of preschool children, since these indices were developed taking into account the specific recommendations for this particular age group.

A diet–lifestyle quality index for young children and its relation to obesity, the “Preschoolers Diet-Lifestyle Index” (PDL-Index), was developed and validated in a sample of 2287 children from the GENESIS cohort [33]. The existing recommendations were incorporated into the PDL-Index and dietary intake data was obtained using the aforementioned combination of techniques [32,33]. Eleven components, including the frequency of consumption of selected foods/food groups, time spent watching television watching and moderate-to-vigorous physical activities, were scored 0 to 4, the total PDL-Index ranging from 0 to 44. The children following healthier diet-lifestyle patterns (third tertile of the PDL-Index) were less likely to be obese/overweight than those following unhealthy diet-lifestyle patterns (first tertile of the PDL-Index) [33]. Despite further modifications which may need to be implemented in order to improve the diagnostic accuracy of the PDL-Index, this was proposed as a tool to be used by healthcare professionals to assess the degree of adherence to specific dietary and lifestyle recommendations and to identify preschool children with increased probability of becoming overweight/obese [33].

#### 3.2.7. Cyprus

The energy density of the dietary intake of children in Cyprus was assessed in an analysis nested within the IDEFICS study [15]. Three-hundred and fifty-one children aged 2–5 years in Cyprus consumed daily mean energy densities of 1.87 Kcal/g exclusively from solid foods and 1.44 Kcal/g from solid foods *plus* energy-containing beverages, in which the mean energy intake from beverages contributed 168 Kcal per day [15].

## 4. Current Economic Crisis and Food Insecurity: An Impact Deserving Investigation

After the economic crisis of 2008, youth unemployment increased sharply in almost all European countries, reaching alarming record levels of more than 50% in Greece and Spain and almost 40% in Italy and Portugal in 2013, with France being an exception with rates only slightly higher than the European Union average [59]. As youth unemployment is a structural indicator of a depressed economy, food insecurity becomes a matter of concern [59]. As a consequence of depression, households are predisposed to less food security with a decrease in the frequency and quantity of food intake for both adults and children [60]. In this context, poor diet in young Greek children was independently associated with maternal unemployment [32].

In response to an enquiry carried out in 2012–2013 in Portuguese healthcare centers nationwide, the Portuguese population stated that the most frequent reason (26.8%) for not always acquiring foods that they wanted or needed was insufficient money [61].

Compared with other regions of Europe, a high prevalence of obesity was found in children aged 2–10 years from southern European, especially from population groups with lower education and income levels [17]. It is controversial whether children from food insecure households are predisposed to being overweight [62]. The association between food insecurity and toddlers being overweight was examined in a North American cohort [62]. After controlling for parental, household, and child characteristics, significant effects of food insecurity on parenting practices were found, which in turn were significantly associated with infant feeding and subsequently toddlers being overweight [62]. Whether the current economic crisis in the Mediterranean countries of the European Union has had a negative impact on the diets and nutritional status of their young infants is a matter for investigation.

## 5. Conclusions

The Mediterranean-like diet is not necessarily associated with living in a Mediterranean region. Analyses from multi-national European studies found low adherence to a Mediterranean-like dietary pattern associated with high prevalence of overweight/obesity in children from the Mediterranean countries of the European Union.

National studies from some countries, such as Portugal, Spain, and Greece, confirm a frequent consumption of fruit and vegetables despite a high prevalence of overweight/obesity in young children. This may be related to extra energy intake from sugared beverages and snacks and higher than recommended protein intake, especially from dairy products. Another matter of concern was the excessive sodium intake found in the great majority of young children. The early consumption of energy-dense foods and overweight seems to track over time. Maternal education and the familial socioeconomic status are associated with better quality diets.

Programs attempting to improve adherence to the traditional Mediterranean diet should be part of a multi-intervention strategy to promote health and to prevent overweight/obesity. These programs should promote the restriction of extra energy and protein from dairy products, as well as energy restriction from foods with saturated fats, sugars, and salt. Whole fresh fruit and vegetables daily-consumption should be promoted, and sugared beverages should be replaced by water. Families, health professionals, and politic stakeholders should be involved as partners, in order to change the described early unhealthy and obesogenic behaviors.

## Figures and Tables

**Table 1:** Multi-national European cohort studies investigating dietary habits, in comparison to the Mediterranean diet-like pattern, and weight status of preschool children, including those living in Mediterranean countries of the European Union.

Author, Year (Reference)	Study Design/Name of the Study	Countries	Age	Sample Size	Outcome	Instruments	Results
De Lauzon-Guillain *et al.*, 2013 [13]	Multi-national European birth cohort study	Four European countries, including the Mediterranean countries France, Portugal, and Greece	2–4 years	British ALSPAC cohort *N* = 7269; French EDEN cohort *N* = 1302; Portuguese Generation XXI cohort *N* = 556; and Greek EuroPrevall cohort *N* = 800	Influence of early feeding practices on later fruit and vegetable intake in preschool children	- FFQ	- Breastfeeding for a longer period after birth was related to higher fruit and vegetable intake. - In the Mediterranean cohorts, the mean fruit and vegetable intakes (servings/day) at 2-year old were respectively: 1.3 and 1.1 in the French cohort, and 1.1 and 0.7 in Greek cohort; and at 4 years old, 1.7 and 3.3 in the Portuguese cohort.
Hebestreit *et al.*, 2014 [14]	Multi-national European cohort study “IDEFICS”	Eight countries, including the Mediterranean countries Italy, Cyprus, and Spain	Subsample 2–5 years	Mediterranean subgroup, *N* = 1583 (Italy 854, Cyprus 419, and Spain 310)	Associations between proxy-reported energy intake, daily food intake, and EDF with BMI z-score	- fMDS - Anthropometry	- Proxy-reporters are subject to misreporting, in particular for children in the higher BMI levels. - Energy intake is a more important predictor of unhealthy weight development in children than the daily food intake.
Hebestreit *et al.*, 2014 [15]	Multi-national European cohort study “IDEFICS”	Eight countries, including the Mediterranean countries Italy, Cyprus, and Spain	Subsample 2–5 years	Mediterranean subgroup, *N* = 1416 (Italy 776, Cyprus 351, and Spain 289)	Description of energy density of dietary intake	- 24-h dietary recalls plus school meal assessments	- In the pre-school Mediterranean children subgroup, the mean food density was 1.81 Kcal/g from exclusively solid foods, and 1.27 Kcal/g from solid foods *plus* energy-containing beverages. - The daily intake from beverages was 284 Kcal.
Tognon *et al.*, 2014 [16]	Multi-national European cohort study “IDEFICS”	Eight countries, including the Mediterranean countries Italy, Cyprus, and Spain	2–9 years	Mediterranean subgroup recalls, *N* = 2675 (Italy 1385, Cyprus 879, and Spain 411)	Adherence to a Mediterranean-like diet	- 24-h dietary recalls on frequency of consumption of 43 foods - fMDS	High adherence to a Mediterranean-like diet in children aged 2–5 years: - Italy: girls 55.0%, boys 55.9% - Spain: girls 37.1%, boys 32.3% - Cyprus: girls 29.5%, boys 33.9%
Tognon *et al.*, 2014 [17]	Multi-national European cohort study “IDEFICS”	Eight countries, including the Mediterranean countries Italy, Cyprus, and Spain	2–9 years	*N* = 9114	Association between Mediterranean-like diet and overweight/obesity	- fMDS - Anthropometry	- High fMDS levels at baseline protected against increases in BMI, waist circumference, and waist-to-height ratio, with a similar trend for percent fat mass. - Parental education and high parental income were determinants of high fMDS levels.
Ahrens *et al.*, 2014 [18]	Multi-national European cohort study “IDEFICS”	Eight countries, including the Mediterranean countries Italy, Cyprus, and Spain	2–10 years	Mediterranean subgroup, *N* = 7551 (Italy 2437, Cyprus 2149, and Spain 2965)	Assessment of prevalence of overweight and obesity in European children	- International Obesity Task Force (2012) weight categories	- Higher prevalence of overweight and obesity was found in the Mediterranean countries, respectively: 25.0% and 17.4% in Italy, 15.5% and 7.2% in Cyprus, and 14.3% and 10.0% in Spain 17. - Overall, population groups with low income and/or lower education levels showed the highest prevalence of obesity.

Legend: BMI body mass index, EDF energy-dense foods, FFQ food-frequency questionnaire, fMDS frequency-based Mediterranean Diet Score.

**Table 2:** National data on dietary habits and weight status of preschool children living in Mediterranean countries of the European Union.

Country	Author, Year (Reference)	Study Design/Name of the Study	Age/Sample Size	Outcome	Tools	Results
Cyprus	Hebestreit *et al.*, 2014 [15]	Multi-national European cohort study “IDEFICS”	Subgroup 2–5 years Cypriot subsample *N* = 351	Associations between proxy-reported energy intake, daily food intake and EDF	- fMDS	Consumption of mean energy densities: - 1.87 Kcal/g exclusively from solid foods. - 1.44 Kcal/g from solid foods *plus* energy-containing beverages; the mean energy intake from beverages contributed with 168 Kcal *per* day.
France	Fantino *et al.*, 2008 [27]	National cross-sectional survey	1–3 years *N* = 706	Assessment of energy and nutrient intake and adequacy of diet	- 3-day weighed food records - Conversion into intakes of energy and nutrients according to food composition databases	- In relation to EAR, the mean daily energy intake was slightly higher up to 7 months of age, and lower after 1-year of age. - Globally, the diet was adequate for a large proportion of children and satisfied most of their nutritional requirements. - Mean daily intake of energy, protein, fat, and carbohydrate were adequate. - Mean daily intake of sodium, calcium, magnesium, phosphorus and B group vitamins were above the DRI. - Intake of alpha-linolenic acid, vitamin E, vitamin C, iron, and zinc was inadequate in some of children over 1-year of age.
	Lioret *et al.*, 2015 [28]	Mother–child cohort “EDEN” cross-sectional study at 2, 3, and 5 years of age *plus* longitudinal study on data collected at these 3 ages	2-years → 5-years *N* = 989	Assessment of dietary patterns at 2, 3, and 5 years of age. Assessment of tracking between patterns. Associations between the dietary patterns and sociodemographic factors	- FFQ	- Dietary patterns identified at 2-years old: “Processed and fast foods”, positively correlated with French fries, processed meat, carbonated soft drinks, chocolate, chips, cookies, pizza, fruit juice, meat, dairy desserts, and ice cream; “Guidelines”, characterized by frequent consumption of cooked vegetables, rice, fresh fruit, raw vegetables, low-fat fish, potatoes, ham, stewed fruit, and meat; and “Baby foods”, positively correlated with baby foods, breakfast cereals, and stewed fruit. - Moderate correlation coefficients were found between similar patterns across the 3 assessed ages. - The “Processed and fast foods” at the 3 ages was inversely associated with maternal education and age, and positively associated with the presence of older siblings. - The “Guidelines” at 2, 3, and 5-years was predicted by maternal education.
Greece	Manios *et al.*, 2008 [9]	Cross-sectional study on a sample of GENESIS cohort	1–5 years *N* = 2374	Assessments of nutrient adequacy of the diets and the nutritional status	- 2-day weighed food records - FFQ - Anthropometry	- In the pooled sample the prevalence of “at risk of overweight” and “overweight” was 16.7% and 16.4%, respectively; and the mean usual energy intake was 117% of the mean estimated energy requirement. - The “at risk of being overweight” and “overweight” children had significantly higher mean daily energy intakes than their normal-weight counterparts. “Overweight” children consumed more energy from total fat and saturated fat and less from carbohydrate. - The usual protein intake was within the AMDR, but 21.0% of children had lower intakes for carbohydrate and 59.5% higher intakes for fat. - The estimated prevalence of inadequacy was between 10% and 25% for niacin, vitamin E and folate, and usual intakes exceeding the tolerable upper intake levels were recorded for Zn and Cu.
	Manios *et al.*, 2009 [32]	Cross-sectional study on a sample of GENESIS cohort	2–5 years *N* = 2287	Assessments of diet quality and socio-demographic factors related to it. Development of the “Healthy Eating Index “	- Weighed food records - 24-h recalls - Food diaries	- About 80% of children had a poor diet (low Healthy Eating Index score), associated with low fruit, vegetable, and grains intake and high saturated fat intake. - Poor diet was associated with lower levels of physical activity, lower maternal educational level, and unemployment status.
	Manios *et al.*, 2010 [33]	Cross-sectional study on a sample of GENESIS cohort	2–5 years *N* = 2287	Development of a diet–lifestyle quality index for young children and its relation to obesity (Preschoolers Diet-Lifestyle Index—PDL-Index)	- Weighed food records - 24-h recalls - Food diaries	- Eleven components were scored 0 to 4, the total PDL-Index ranging from 0 to 44. - Children following healthier diet-lifestyle patterns (third tertile of the PDL-Index) were less likely to be obese/overweight than those following unhealthy diet-lifestyle patterns (first tertile of the PDL-Index).
Italy	Verduci *et al.*, 2007 [29]	Single-center birth cohort	1-year → 10-years *N* = 61	Assessment of dietary energy and macronutrient intakes and comparison with recommended dietary allowances	- FFQ	At preschool ages - At 1-year old: the mean total daily energy intake was 94 Kcal/Kg; the mean %TEV were 48.3% from carbohydrate, 33.6% from lipids, and 20.4% from protein. - At 5-years old: the mean total daily energy intake was 119 Kcal/Kg; the mean %TEV were 57% from carbohydrate, 32% from lipids, and 15% from protein. - Compared with the Italian recommended dietary allowances, the protein intake was high at any age analyzed and the energy intake was high at 5-years. - In the longitudinal pattern, boys had significantly higher energy intake and glycemic load.
	Hebestreit *et al.*, 2014 [15]	Multi-national European cohort study “IDEFICS”	Subgroup 2–5 years Italian subsample *N* = 776	Associations between proxy-reported energy intake, daily food intake and EDF	- fMDS	Consumption of mean energy densities: - 2.24 Kcal/g exclusively from solid foods. - 1.61 Kcal/g from solid foods *plus* energy-containing beverages; the mean energy intake from beverages contributed with 208 Kcal *per* day.
	Zuccotti *et al.*, 2014 [30]	Cross-sectional study “NUTRINTAKE”	6 Months–3 years *N* = 390	Comparison of the dietary intake between children living in North and South Italy	- 7-day weighed food records - Anthropometry	- In the pooled sample: normal anthropometry and energy intake were found, despite high intake of proteins, simple carbohydrates, saturated fats, and sodium, and low intake of iron and fiber. - Anthropometry, energy, and macronutrient intakes were similar in North and South Italy; in North, iron intake was 27% lower and fiber intake 16% higher than in South.
Malta	Buttigieg *et al.*, 2012 [31]	Cross-sectional study	3-years *N* = 200	Associations between: - Parenteral and childhood obesity - Maternal awareness of public health promotion on healthy eating and parenteral and childhood obesity	- Face-to-face interviews - Anthropometry	- Significant association was found between childhood obesity and obesity in either of the parents. - Full maternal awareness of healthy eating promotion appeared to have a protective effect against the development of childhood obesity/overweight.
Portugal	Rêgo *et al.*, 2013 [19]	Cross-sectional national study “EPACI”	*N* = 2232	Evaluation of feeding pattern and weight status	- FFQ - 3-days food diary - Anthropometry	- Daily intakes were: fresh fruit 93%, vegetables 99%, sugared beverages 17%, and sugared desserts 10%. - Mean daily energy intake was 1200 Kcal; %TEV from carbohydrates 49.9%, from fat 28.5%, and from protein 18.2%. The foods mostly contributing to total energy intake were dairy products (28%), cereals (15%), and meat (13%). - Mean daily protein intake was 4.5 g/Kg. The foods mostly contributing to total protein intake were dairy products (36%), meat (28%), and fish (13%). - Mean daily sodium intake was 1843 mg; 87.3% of children exceeded the upper recommended limit. - Absence of inadequacy except for vitamin E (all sample), and folate and B1 at 12–23 months - Prevalence of overweight 31.4% with 6.5% of obesity
	Lopes *et al.*, 2014 [21]	Cross-sectional regional study, on a sample of Geração XXI birth cohort	*N* = 8647	Evaluation of feeding pattern and weight status	- FFQ and food diaries - Anthropometry	- Daily consumption was: fruits 86%, vegetables 92%, cakes and candies 65%, and sugared beverages 52%; 73% consumed salty snacks 1–4 times/week. - Main sources of saturated fatty acids were dairy products 33.4%, meat 20.7%, and savory pastries and sugared desserts 14.7%. - Excessive sodium intake was found in 99%. - Insufficient intakes were found for vitamin D in 100%, vitamin E in 98.6%, folate in 21.5%, and calcium in 7.0%. - Prevalence of overweight was 13.8%, and obesity 6.2%.
	Vilela *et al.*, 2015 [22]	Cross-sectional regional study, on a sample of Geração XXI birth cohort	*N* = 9114	Association between socioeconomic characteristics and family structure and consumption of EDF	- FFQ - EDF groups: soft drinks; sweets; cakes; and salty snacks	Lower EDF consumption was associated with: - High socioeconomic characteristics. - Influence from parents’ background and from the preceding generations. - Absence of older siblings.
	Vilela *et al.*, 2014 [23]	Longitudinal regional study, using a sample of Geração XXI birth cohort	*N* = 705	Association between the consumption of EDF at 2 years old and the consumption of foods and diet quality at 4 years old	- FFQ - EDF groups: soft drinks; sweets; cakes; and salty snacks - Healthy eating index for 4 years old children	- The consumption of EDF at 2-years old was negatively associated with the diet quality at 4-years old. - Higher intakes of soft drinks, salty snacks, and sweets at 2-years old were associated with lower consumption of fruit and vegetables at 4 years old.
	Durão *et al.*, 2015 [24]	Longitudinal regional study, on a sample of Geração XXI birth cohort	*N* = 589	Association between consumption of EDF at 2 years old and BMI at 4 years old	- FFQ - Anthropometry - Cross-lagged panel design	- No association was found between consumption of EDF at 2-years old and BMI at 4-years old. - Both the consumption of EDF and BMI z-scores tracked across toddler and preschool ages.
	Durão *et al.*, 2015 [25]	Cross-sectional regional study on a sample of Geração XXI birth cohort	*N* = 4122	Association between maternal perceived responsibility and child feeding practices and dietary inadequacy	Mothers self-completed: - The Child Feeding Questionnaire - A scale on covert and overt control	- Children whose mothers had higher levels of covert control, monitoring, and restriction were less likely to consume fruits and vegetables below recommendations and EDF above tolerable limits. - Higher pressure to eat was associated with higher possibility of children consuming dairy above recommendations.
Spain	Dalmau *et al.*, 2015 [26]	Cross-sectional study “ALSALMA”	*N* = 1701	Comparison of the nutritional patterns with the recommendations	- Dietary diary on food intake on 4 non-consecutive days	- Mean energy intake was 123%–124% higher than the DRI for 13–36 months of age. - Mean percentage of protein intake increased with age, being 370% and 441% higher than the adequate intake for 13–24 months and 25–36 months of age, respectively. - Higher percentages in daily intake of proteins and carbohydrates and lower percentage of total lipids were significantly related to a greater BMI, regardless of energy intake. - Proportions of children with deficient micronutrient intakes for 13–24 months and 25–36 months of age were respectively: 81.7% and 92.1% for vitamin D; 39.3% and 53.4% for vitamin E; 12.5% and 14.8% for folic acid; 10.1% and 5.5% for calcium; and 27.1% and 31% for iodine.
	Hebestreit *et al.*, 2014 [15]	Multi-national European cohort study “IDEFICS”	Spanish subsample *N* = 298	Associations between proxy-reported energy intake, daily food intake and EDF	- fMDS	Consumption of mean energy densities: - 1.67 Kcal/g exclusively from solid foods - 1.16 Kcal/g from solid foods *plus* energy-containing beverages; the mean energy intake from beverages contributed with 307 Kcal *per* day

*Legend*: %TEV percentage of total energy value; AMDR acceptable macronutrient distribution ranges; BMI body mass index; DRI daily recommended intakes; EAR estimated average requirement; EDF energy-dense foods; FFQ food-frequency questionnaire; FFQ food-frequency questionnaire; fMDS frequency-based Mediterranean Diet Score; PDL-Index Preschoolers Diet-Lifestyle Index.

## Abbreviations

The following abbreviations are used in this manuscript:

ALSALMA	ALimentando la SAlud del MAñana
ALSPAC	Avon Longitudinal Study of Parents and Children
AMDR	acceptable macronutrient distribution ranges
BMI	body mass index
DRI	dietary reference intakes
EAR	estimated average requirement
EDEN	Étude des Déterminants pré-et postnatals précoces du développement et de la santé de l’ENfant
EDF	energy-dense foods
EPACI	Estudo do Padrão Alimentar e de Crescimento Infantil
FFQ	food-frequency questionnaire
fMDS	frequency-based Mediterranean Diet Score
GENESIS	Growth, Exercise and Nutrition Epidemiological Study in preSchoolers
HEI	Healthy Eating Index
IDEFICS	Identification and Prevention on Dietary and life style induced health effects in children and infants
IOTF	International Obesity Task Force
PDL-Index	Preschoolers Diet-Lifestyle Index
RDA	recommended dietary allowances
